# Transcranial Magnetic Stimulation Shows Promising Results for Memory and Mood

**DOI:** 10.1002/alz70858_097812

**Published:** 2025-12-24

**Authors:** Jeffrey Shenfeld

**Affiliations:** ^1^ The Memory Center, Englewood, NJ, USA

## Abstract

**Background:**

Transcranial magnetic stimulation (TMS) leads to significant improvement in memory and mood in dementia patients

**Method:**

TMS therapy was initiated in a two separate 75 year old females with advanced Alzheimer's dementia and accompanying depression. Both subjective and objective improvement were noted in both patients based on clinical examinations and diagnostic evaluations.

**Result:**

The first patient described drastic improvements in mood, sleep, appetite, and overall wellbeing. Montreal Cognitive Assessment (MOCA) score improved from a baseline of 16 to an improved score of 27. Similarly, the second patient, described decreased anxiety, improved sleep and a renewed sense of life. Her MOCA improved from a baseline of 17 to 24.

**Conclusion:**

TMS should be strongly considered alone as a noninvasive therapy for dementia patients, or in conjunction with pharmacologic therapy in dementia patients, particularly with comorbid depression.

